# Highly Efficient Removal of PFAS from Water Using Surface-Modified Regenerable Quaternized Chitosan Hydrogels

**DOI:** 10.3390/gels12010014

**Published:** 2025-12-24

**Authors:** Mohammad Bagheri Kashani, Lingfei Fan, Weile Yan, Bridgette M. Budhlall

**Affiliations:** 1 Department of Plastics Engineering, University of Massachusetts, Lowell, MA 01854, USA; 2 Department of Civil and Environmental Engineering, University of Massachusetts, Lowell, MA 01854, USA

**Keywords:** per- and polyfluoroalkyl substances, water treatment, PFAS removal, bio-based chitosan hydrogels, regenerable adsorbents

## Abstract

In this study, surface-modified bio-based hydrogels derived from crosslinked quaternized chitosan (MQCGs) were developed to treat PFAS-contaminated water. The novelty of this work lies in the surface modification and engineering of the hydrogels to enhance the surface area and positive charge of the hydrogels through sacrificial templating. By blending the chitosan solution with polyethylene glycol (PEG) and then removing PEG via sacrificial templating, microscale channels were created on the surface of the hydrogels. This increased the availability of the hydrogel’s positive charges for increased electrostatic interactions with PFAS, achieving >98% PFOS (a long-chain PFAS) adsorption in less than 30 min. Batch adsorption experiments demonstrated that surface-modified quaternized chitosan hydrogels (MQCGs) removed both long- and short-chain PFAS across a pH range of 3 to 12, maintaining their performance over 10 regeneration cycles. The adsorption behavior followed the Freundlich isotherm model and pseudo-second-order kinetics, indicating fast multilayer adsorption on heterogeneous active sites via the combined actions of electrostatic, hydrophobic, and physical interactions. Using PFOS and PFOA as model long-chain PFAS and PFBS and PFHxA as short-chain surrogates, respectively, MQCGs achieved a complete removal of PFOS and PFOA and over a 99.9% removal of PFBS and PFHxA, each at a low concentration of 500 µg/L in water. Moreover, MQCGs exhibited highly efficient removal of PFAS at environmentally relevant concentrations of 20 µg/L in tap water containing MgSO_4_ and NaCl as competing electrolytes, demonstrating the potential of MQCGs as a new class of efficient, selective, and regenerable materials for PFAS sequestration.

## 1. Introduction

Per- and polyfluoroalkyl substances (PFASs), often termed ‘forever chemicals’, are a class of synthetic compounds that possess at least one fluorinated carbon atom in -CF_2_ or -CF_3_ forms [1,2,3]. Owing to their low molecular polarity, strong C-F bond, and high chemical and thermal stability, they are widely used in food packaging, firefighting foams, water-repellent fabric coatings, and pesticides [4,5,6].

Short-chain PFASs are defined as perfluoroalkyl carboxylic acids (PFCAs) containing seven or fewer carbons and perfluoroalkane sulfonic acids (PFSAs) containing six or fewer carbons, with compounds above these limits classified as long-chain PFASs [7]. Short-chain PFAS have increasingly replaced long-chain forms following regulatory restrictions on legacy long-chain PFAS use. However, recent evidence indicates that these alternatives may also present long-term environmental risks [8,9,10].

The disposal of PFAS-containing residues and industrial wastewaters in landfills can lead to PFAS leaching into groundwater and drinking water sources [11,12,13,14]. Exposure to PFAS, especially non-polymeric compounds, is associated with elevated risks of cancer, diabetes, immune dysfunction, and endocrine disruption [15,16]. Although safer alternatives are being actively explored [17,18,19], PFCAs and PFSAs (short- and long-chain) remain the most frequently detected PFAS in the environment [20,21,22,23,24].

Several water treatment techniques have been explored, such as adsorption, ion exchange, reverse osmosis (RO), nanofiltration (NF), and oxidation processes [25,26]. Among them, adsorption is the most cost-effective method and has shown promising results for the removal of PFAS from water sources [27]. It is worth noting that adsorption as a separation technique requires proper downstream processing to prevent secondary contamination, a critical step that must be considered to mitigate the risk of secondary environmental contamination.

Although various adsorbents, including granular/powdered activated carbon (GAC/PAC), biomaterials, metal–organic frameworks (MOFs), and polymer-based synthetic sorbents [28], have been introduced for water treatment applications, several limitations still persist. One challenge is that short-chain PFAS are more difficult to remove via many adsorbents compared to long-chain PFAS [29]. For instance, GAC effectively removes long-chain PFAS but performs poorly for short-chain PFAS (e.g., Perfluorobutanesulfonate (PFBS) and Perfluorohexanoic acid (PFHxA)). Moreover, its regeneration is costly and incomplete, often leading to rapid breakthroughs and reduced long-term efficiency [30]. On the other hand, using nanoparticle adsorbents or MOFs such as ZIF-8 involves complex synthesis procedures and high costs, limiting their large-scale applicability [31].

Recently, chitosan biosorbents have attracted attention in relation to water treatment applications. Chitosan, a poly(*d*-glucosamine) produced by the deacetylation of chitin and the second most abundant biopolymer, possesses a highly reactive amine group [32,33,34]. It has demonstrated promise for removing contaminants such as metal ions, phosphates, and nitrates from water [35]. However, its use for PFAS removal is limited by its poor water stability and contaminant selectivity as well as its pH dependence [36].

Existing chitosan-based sorbents, whether simple crushed particles or crosslinked hydrogels, rely heavily on the protonation of the amine group in acidic conditions, resulting in pH dependence and an inherently low removal efficiency for short-chain PFAS [37]. While modifications like grafting or crosslinking have been attempted to enhance these chitosan-based sorbents’ performance, these methods often suffer from the use of toxic chemicals (e.g., epichlorohydrin), involve complex synthesis processes (chemical grafting of polyether imide on chitosan backbone), or fail to achieve efficient, broad-spectrum removal across wide pH ranges, often limiting testing to long-chain PFAS under acidic conditions [38,39].

Additionally, the low surface area of traditional chitosan hydrogels limits hydrophobic and physical interactions, resulting in the minimal adsorption of PFAS at higher pH levels, where electrostatic interactions are insignificant [28,40].

To enhance the surface area, Zhang et al. [41] studied the adsorption of PFOA through a mixture of covalent organic frameworks (COFs)/chitosan particles. Their study reported that hydrogen bonding between PFOA and chitosan’s hydroxyl groups enhanced the adsorption. However, the adsorption tests were performed only on long-chain PFOA under acidic conditions (at pH~5), and no regenerability study was conducted on the particles. Moreover, using MOFs and COFs in PFAS removal involves insufficient hydrolytic stability, high fabrication complexity and costs, and vulnerability to competitive adsorption from co-contaminants in water [42,43].

To the best of our knowledge, no chitosan-based adsorbent has demonstrated the ability to remove both long-chain and short-chain PFAS effectively and regeneratively in the presence of co-ion contaminants in water.

To address this above-mentioned deficiency, we present an environmentally sustainable method for synthesizing chitosan hydrogels that are surface-modified and quaternized to create regenerable, highly efficient PFAS adsorbents effective across a wide pH range. The novelty of the current work lies in achieving PFAS adsorption without relying on complicated synthesis procedures, such as grafting, or advanced nanomaterials, such as MOFs or COFs.

We instead use a simple, cost-effective surface engineering approach on crosslinked chitosan hydrogels to create microscale surface channels. This modification maximizes the positive charge for high electrostatic and physical interactions with PFAS. We also introduce permanent positive charges on the hydrogels to maintain high activity across a wide pH range. This is unlike typical chitosan adsorbents, which rely on the protonation of amino groups and are thus only effective in acidic conditions. Furthermore, a simple ion exchange mechanism allows for easy regeneration aimed at reusability.

**Figure 1:**
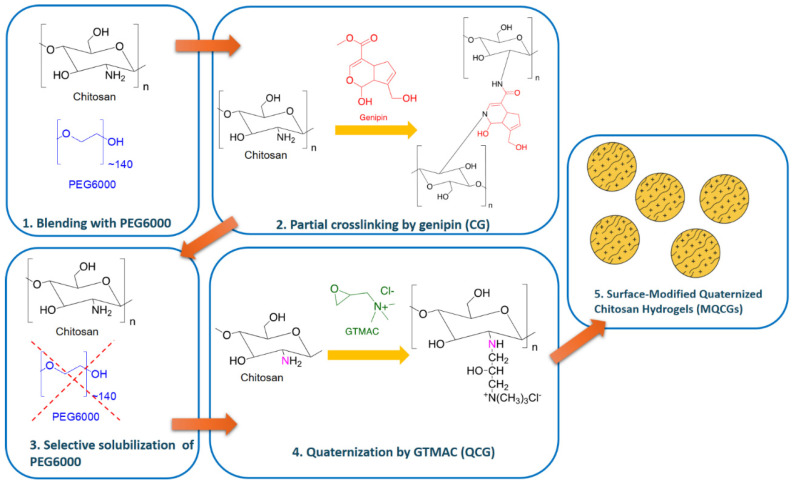
Schematic representing the chemical reactions used to synthesize the surface-modified quaternized chitosan hydrogels (MQCGs).

Initially, chitosan hydrogels (CGs) were first crosslinked with genipin, a biodegradable, plant-derived crosslinker, to improve its aqueous stability. To increase the surface area of the hydrogels, surface channels were created by blending the chitosan solution with a low-molecular-weight PEG and then selectively dissolving the PEG to expose the channels. To support a stable adsorption performance across a varying pH range and in the presence of ionic co-solutes, a permanent positive charge was introduced using glycidyl trimethyl ammonium chloride (GTMAC), yielding the surface-modified quaternized chitosan hydrogel (MQCG) (Figure 1).

The central hypothesis of this study is that using a sacrificial templating process to generate microscale channels on the surface of MQCGs will significantly enhance the available surface area and positive charge density for adsorption. The efficacy and efficiency of the synthesized MQCGs for removing both long- and short-chain PFAS in the presence of competing co-ions in water was evaluated. The influence of the molecular weight and concentration of PEG on the surface structure, morphology, and surface area of the MQCGs was examined. The PFAS removal efficiency, adsorption kinetics, isotherms, and thermodynamics were analyzed to understand the underlying mechanism.

Finally, we evaluated a simple regeneration process using a NaCl solution to investigate whether the MQCG is a potential sustainable and reusable material for PFAS removal from water.

## 2. Results and Discussion

### 2.1. Characterization of Hydrogels

#### 2.1.1. Fourier Transform Infrared–Attenuated Total Reflection Spectroscopy

**Figure 2:**
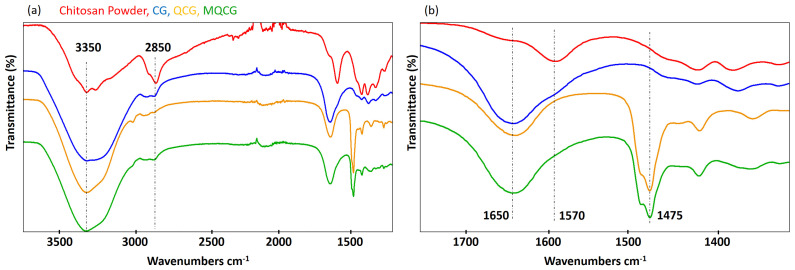
FTIR-ATR spectra of chitosan powder, CG, QCG, and MQCG, at wavenumbers (**a**) 1400–3600 cm^−1^ and (**b**) 1300–1700 cm^−1^.

A Fourier Transform Infrared–Attenuated Total Reflection (FTIR-ATR) spectroscopic analysis was performed to confirm the crosslinking and quaternization of the chitosan hydrogels (Figure 2). Chitosan powder is characterized by a primary amine group peak at 1570 cm^−1^, stretching peaks of CH_3_ in the backbone at 2850 and 2950 cm^−1^, and a broad peak representing hydroxyl groups at 3350 cm^−1^ [44,45].

Crosslinking chitosan with genipin after hydrogel formation results in a shift in the primary amine peak (1570 cm^−1^) to a higher wavelength, transforming it into a secondary amine, which appears at 1650 cm^−1^. A similar shift is observed in QCGs, where the reaction between the glycidyl groups of GTMAC and the primary amine of chitosan leads to the formation of a peak at 1650 cm^−1^ [46]. The peak at 1475 cm^−1^, observed in both the QCG and MQCG, is attributed to the presence of methyl groups introduced during the quaternization of chitosan with GTMAC, confirming the reaction between GTMAC and chitosan [47].

Eventually, the peaks at 3350 cm^−1^ exhibit higher intensity in the CG, QCG, and MQCGs compared to the raw chitosan powder, which can be attributed to the presence of additional hydroxyl groups introduced by the genipin crosslinking in those hydrogels [46].

#### 2.1.2. Microscopy and Elemental Analysis

**Figure 3:**
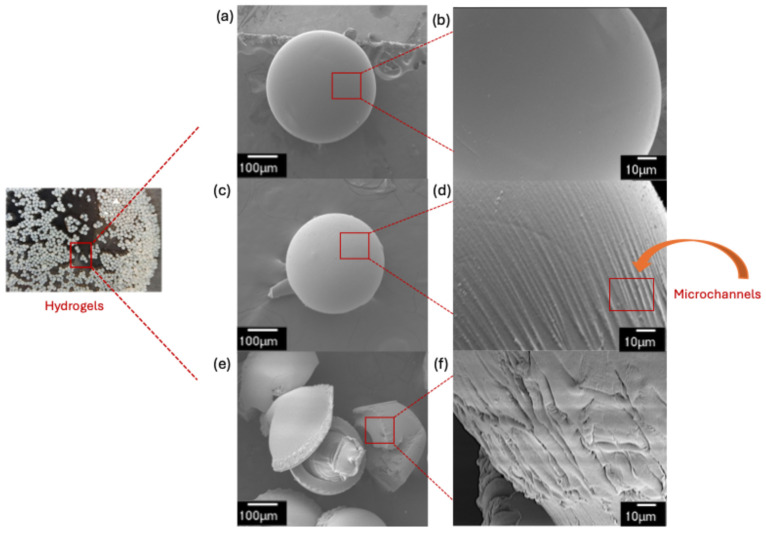
SEM images of (**a**) CG ×250 mag., (**b**) CG ×2000 mag., (**c**) MQCG1 ×250 mag., (**d**) MQCG1 ×2000 mag., (**e**) MQCG2 ×250 mag., and (**f**) MQCG2 ×2000 mag.

Scanning electron microscopy (SEM) was used to evaluate the surface morphology of the chitosan hydrogels before and after incorporating PEG-6000. Figure 3 presents SEM images of the CG, MQCG1, and MQCG2.

The spherical morphology of the CG and MQCG1 is evident in Figure 3a,c. Increasing the magnification from ×250 to ×2000 reveals the surface morphology of the hydrogels. As depicted in Figure 3b, the surface of the chitosan hydrogels before the incorporation of PEG-6000 is smooth. However, adding 1 wt.% PEG-6000 to the chitosan solution, followed by its removal using a water/ethanol mixture, results in the formation of surface channels (Figure 3d).

Increasing the PEG content from 1 wt.% to 2 wt.% leads to MQCG2’s formation (Figure 3e,f), characterized by a coarser surface with deeper channels. Additionally, the extraction of a higher amount of PEG-6000 after the hydrogel formation disrupts the spherical morphology and reduces the mechanical integrity of the hydrogels due to deep craters and the possibility of internal pore formation [48].

At higher PEG concentrations, the hydrogels’ ability to withstand handling or multiple cycles is lost because the large volume of the sacrificial template creates excessive void space, rendering the swollen hydrogel fragile and prone to fragmentation during handling and regeneration (see Figures S1 and S2 in the Supporting Information).

**Figure 4:**
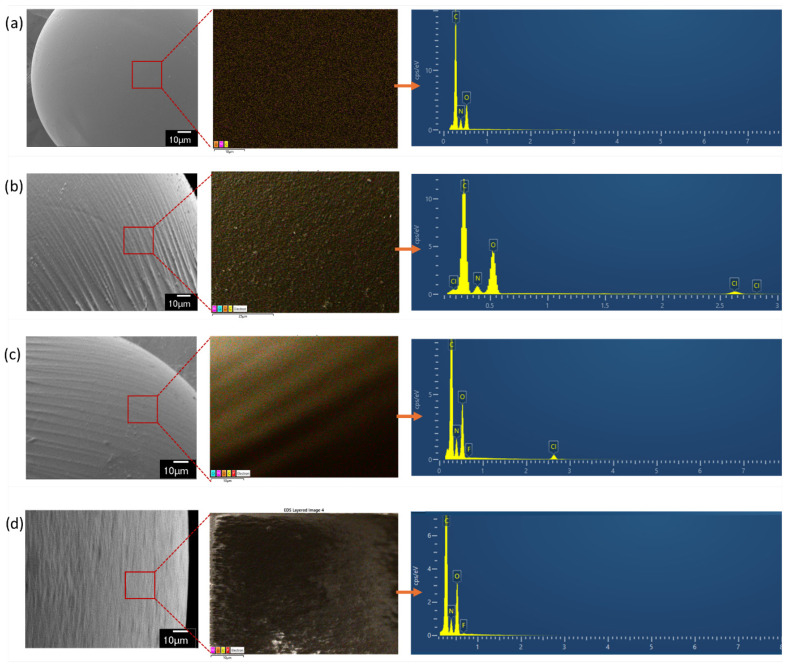
Electron dispersive spectra of (**a**) CG, (**b**) MQCG, (**c**) MQCG after 30 min of PFBS adsorption, and (**d**) MQCG after 24 h of PFBS adsorption.

An energy-dispersive spectroscopy (EDS) analysis was performed to examine the elemental composition of CGs before the quaternization and surface modification (Figure 4a), the MQCG after quaternization with GTMAC (Figure 4b), and the MQCG after 30 min (Figure 4c) and 24 h (Figure 4d) of PFBS adsorption. The elemental mapping and spectrum of the CG (Figure 4a) demonstrate the presence of carbon, nitrogen, and oxygen, which correspond to the chitosan backbone [49].

The surface modification does not affect the elemental composition of chitosan hydrogels, as confirmed by the thorough washing of PEG-6000 with ethanol/water solutions. However, after the quaternization with GTMAC, chlorine atoms appear in the elemental mapping and spectrum of the MQCG alongside carbon, nitrogen, and oxygen (Figure 4b).

Following PFBS adsorption, fluorine atoms are detected in the elemental spectra of the MQCG at both 30-min and 24-h intervals (Figure 4c,d). Notably, after 24 h of the adsorption experiment, the chlorine peaks disappear from the elemental spectrum, likely due to the complete consumption of protonated amine active sites for the PFBS adsorption on the MQCG.

#### 2.1.3. Zeta Potential Measurements

**Figure 5:**
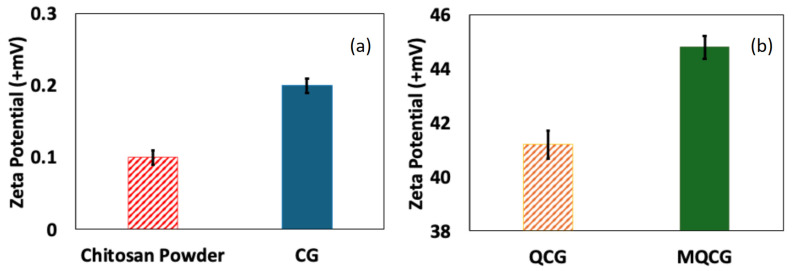
Zeta potential of (**a**) chitosan powder and CG, (**b**) QCG, and MQCG. (Error bars represent the standard deviation of at least five replicates for each data point).

The zeta potential of the hydrogels was measured to evaluate the surface charge generated on the, with the results presented in Figure 5 (see Figure S4 in the Supporting Information for the raw data plots). The chitosan powder and CGs (Figure 5a,b) exhibit near-zero positive charges (0.1 mV and 0.2 mV, respectively), indicating that the amine groups are either unprotonated or only slightly protonated due to the pH of the DI water being slightly acidic (below 7) [50]. However, after the quaternization with GTMAC, the zeta potential of the hydrogels increases significantly to approximately +41.2 mV. This increase is attributed to the permanent protonation of the amine groups following the quaternization reaction [50].

Furthermore, the zeta potential of the MQCGs is slightly higher than that of QCGs (+44.8 mV vs. +41.2 mV). This difference is due to the increased surface area of the MQCGs, which enhances the accessibility of the primary amine groups, allowing for more extensive quaternization and formation of tertiary ammonium groups [51]. The surface area enhancement followed by an increased positive charge after the sacrificial templating of PEG is confirmed by zeta potential measurements and discussed further in Section 2.1.5.

#### 2.1.4. Thermogravimetric Analysis

**Figure 6:**
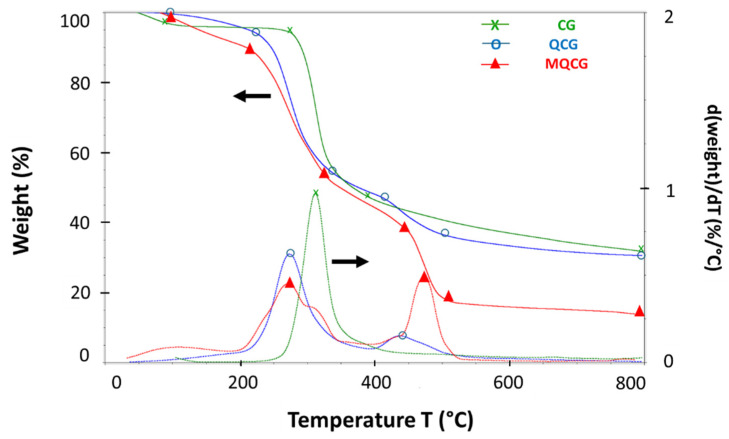
Thermogravimetric/temperature derivative (TGA/DTG) graphs of CG, QCG, and MQCG.

The thermal stability and degradation steps of the hydrogels under N_2_ are shown in Figure 6. As observed in the thermogram of the CGs (green curves), there is an approximate 5% weight loss that occurs between the temperatures of 50 °C and 120 °C, which is due to the evaporation of water that is already within the polymer structure. This amount of water loss is typical for chitosan hydrogels, as reported previously [52].

The major weight loss of the chitosan hydrogels, CGs, (~50.8%) occurred between 200 °C and 400 °C, with the peak weight loss at 300 °C, as demonstrated by the CG’s temperature derivative (DTG) curve. This mass loss could be attributed to the degradation of the polysaccharide backbone of the molecule [53,54]. The char residue for the CGs is ~35%.

The quaternized chitosan hydrogels, QCGs (blue curves), exhibit multiple weight loss steps. The first weight loss step of 8% is due to water evaporation observed between 50 °C and 120 °C within the QCGs. The major weight loss (45% between 250 °C and 350 °C), attributed to the degradation of the polysaccharide backbone, begins at a lower temperature (250 °C) compared to the CGs (300 °C). This decrease in thermal stability may be due to the attachment of the genipin crosslinking agent and the GTMAC quaternizing agent to the primary amine groups of the chitosan backbone, which reduces intermolecular hydrogen bonding in the chitosan molecules [55].

It was previously found that the reduction in hydrogen bonding lowers the thermal stability of the QCGs compared to CGs [46,56]. Further degradation is observed in the QCG compared to the CGs, which occurs between 400 °C and 500 °C (10% weight loss). These additional peaks above 400 °C are absent in CGs, strongly suggesting that they originate from the decomposition of the crosslinked network. In crosslinked chitosan, certain thermally stable structures do not degrade during the primary chitosan decomposition stage (250–350 °C) but instead decompose at higher temperatures (~400–600 °C) [57,58].

Similar thermal behaviors are observed for MQCGs (red curves) compared to QCGs. After the removal of intermolecular moisture trapped in the hydrogels at temperatures below 120 °C (~8% weight loss), the major thermal degradation step begins at lower temperatures compared to chitosan hydrogels, with the major weight loss (~42%) resulting from the polysaccharide backbone breakage occurring between 200 °C and 300 °C [57].

The degradation observed between 400 °C and 500 °C is attributed to the decomposition of crosslinked sections that degrade at higher temperatures. A lower char residue is observed for MQCGs, indicating reduced thermal stability compared to QCGs and CGs. This can be attributed to the increased surface area caused by the formation of microscale surface channels [58]. This increased surface area accelerates the thermal decomposition of MQCGs relative to QCGs and CGs at elevated temperatures. However, this property does not affect the functionality of MQCGs, as their intended water filtration application is designed for room-temperature conditions [38].

#### 2.1.5. BET Surface Area Measurements

**Figure 7:**
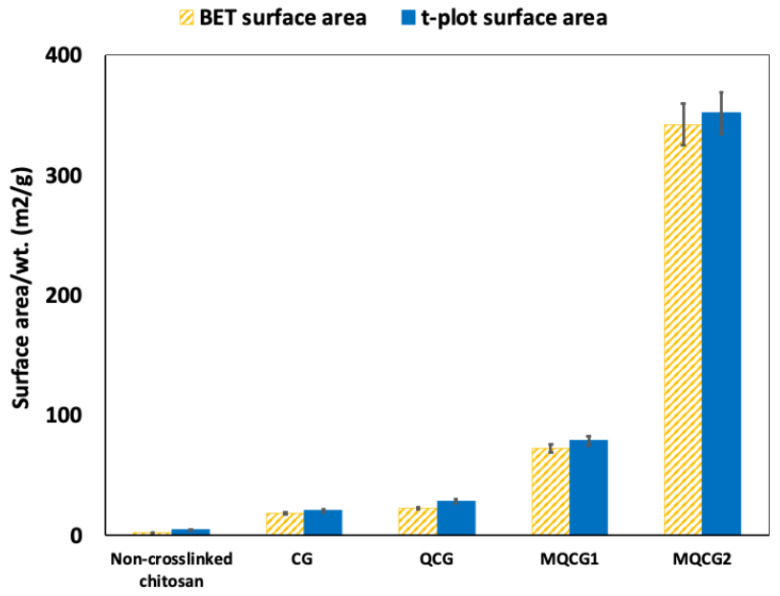
BET (striped, orange) and *t*-plot external surface area (blue) of non-crosslinked chitosan, CG, QCG, MQCG1, and MQCG2. (Error bars represent the standard deviation of at least five replicates for each data point.).

Figure 7 compares the surface areas of chitosan hydrogels before and after crosslinking, as well as QCGs and MQCGs. The *t*-plot method often yields slightly higher surface area values than the BET due to its ability to account for micropore filling at low relative pressures, which is not fully captured by the BET model [59]; hence, both methods were performed to compare the hydrogel results.

As illustrated in Figure 7, non-crosslinked chitosan hydrogels demonstrate a BET and *t*-plot surface area of 2.24 ± 0.09 m^2^/g and 4.41 ± 0.18 m^2^/g, respectively. This small surface area, which corresponds to their low nitrogen adsorption capacity, may be due to their smooth surface before crosslinking and the generation of surface microchannels [48].

Consequently, crosslinking the hydrogels with genipin in CGs increased the BET and *t*-plot surface area to 18.52 ± 0.74 and 20.45 ± 0.82 m^2^/g, respectively. This increase might be a result of the surface micropores that can occur in the crosslinking reactions [60]. Moreover, crosslinking the chitosan hydrogels can lead to a decrease in the diameter of the hydrogels, leading to an increased surface area to volume ratio [44,58].

QCGs demonstrate surface areas comparable to those of the crosslinked hydrogels, albeit slightly higher. Their BET and *t*-plot surface areas are 22.42 ± 0.91 and 28.54 ± 1.14 m^2^/g, respectively. This increase is attributed to the quaternization process, which may introduce defect sites and micropores within the chitosan framework, therefore enhancing the available surface area [61].

A significant increase in surface area is observed in MQCG1, with BET and *t*-plot surface areas of 72.43 ± 2.91 and 78.92 ± 3.16 m^2^/g, respectively. The surface area of MQCG1 is enhanced by the formation of multiple microchannels on the hydrogels’ surfaces, as shown in Figure 3. The increased surface area leads to a higher number of reactive sites, resulting in an increase in the positive charge after quaternization, as confirmed by the zeta potential analysis (Figure 5) [51].

The BET and *t*-plot results of the MQCG2 sample with 2 wt.% PEG-6000 demonstrate significantly higher surface areas compared to MQCG1 (342.12 ± 13.68 and 351.68 ± 14.07 m^2^/g). This drastic enhancement is presumed to result from the bulk porosity introduced through the sacrificial templating of PEG, leading to the formation of internal pores throughout the hydrogel’s bulk structure. These internal pores facilitate the passage of N_2_ gas during the BET analysis [62].

In other words, the large surface area of MQCG2 is not only attributed to the microchannels on its surface, as seen for MQCG1, but is also due to its high internal porosity [62]. These hydrogels, as shown in Figure 3, break easily due to their highly porous structure and are not suitable for the regenerative desorption of the PFAS [63]; hence, they were not studied further [51] (see Section S3 in the Supporting Information and Figure S3).

### 2.2. PFAS Adsorption Performance

#### 2.2.1. Removal Efficiency and Adsorption Capacity at Equilibrium

**Figure 8:**
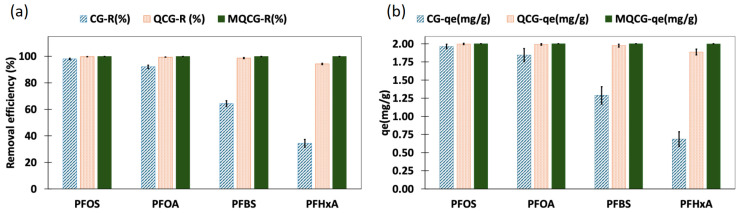
(**a**) The removal efficiency (%) and (**b**) adsorption capacity at equilibrium, *q_e_* (mg/g), of CGs, QCGs, and MQCGs for long-chain (PFOS and PFOA) and short-chain (PFBS and PFHxA) PFAS (Error bars represent the standard deviation of triplicate measurements for each data point).

The removal efficiency (%) and equilibrium adsorption capacity (mg/g) of the hydrogels for different PFAS (short- and long-chain) were evaluated to examine the effects of the quaternization and surface modification (Figure 8). As shown in Figure 8a, the chitosan hydrogel (CG) exhibited high removal efficiency for long-chain PFAS, achieving ~98% for PFOS and ~92% for PFOA. However, its performance declined markedly for short-chain PFAS, with only a ~64% and ~34% removal for PFBS and PFHxA, respectively.

This reduced affinity for short-chain PFAS results from the limited protonation of primary amine groups at a neutral pH and the weaker hydrophobic interactions associated with shorter perfluoroalkyl tails [41]. This is similar to the limitations observed for the short-chain PFAS adsorption by GAC materials [64]. In contrast, both the QCG and MQCG demonstrate significantly improved adsorption for both long-chain and short-chain PFAS. The enhanced performance of QCGs is attributed to the introduction of permanent positive charges, which improve electrostatic interactions between anionic PFAS and these hydrogels [61].

As shown in Figure 8a, QCGs achieved removal efficiencies comparable to MQCGs for long-chain PFAS (99.84% and 99.48% versus 100% for PFOS and PFOA, respectively), but exhibited lower removal rates for short-chain compounds. QCGs removed 98.7% of PFBS and 94.3% of PFHxA, whereas MQCGs achieved 99.98% and 99.96% removal for the same PFAS. The same pattern was reflected in the $q_{e}$ values (Figure 8b): the corresponding equilibrium adsorption capacities of CGs are 1.962 and 1.840 mg g^−1^ for PFOS and PFOA, with markedly lower values of 1.291 and 0.691 mg g^−1^ for PFBS and PFHxA. QCGs demonstrated $q_{e}$ values of 1.997 (PFOS), 1.980 (PFOA), 1.974 (PFBS), and 1.884 (PFHxA) mg g^−1^, while for MQCGs, the introduction of surface microchannels increased both the available surface area and the density of positive sites, enhancing the $q_{e}$ values for both long-chain and short-chain PFAS [65]. Therefore, MQCGs outperformed both CGs and QCGs for all PFAS tested. Table 1 summarizes the adsorption results for all hydrogels for different PFAS types at 500 µg/L, measured at equilibrium.

The notation of ≥2.000 mg g^−1^ for the *q_e_* of MQCGs is used because these hydrogels drove effluent concentrations below the analytical Limit of Quantification (LOQ). This 2 mg/g value represents the measurable capacity ceiling for the current experimental parameters (C_0_, V, and M). It should be noted that this does not affect the relative performance comparison between different hydrogels, as the superior removal efficiency of the MQCG is already established before the analytical limit is reached.

#### 2.2.2. The Isotherm and Kinetics of the Adsorption of PFOS

**Figure 9:**
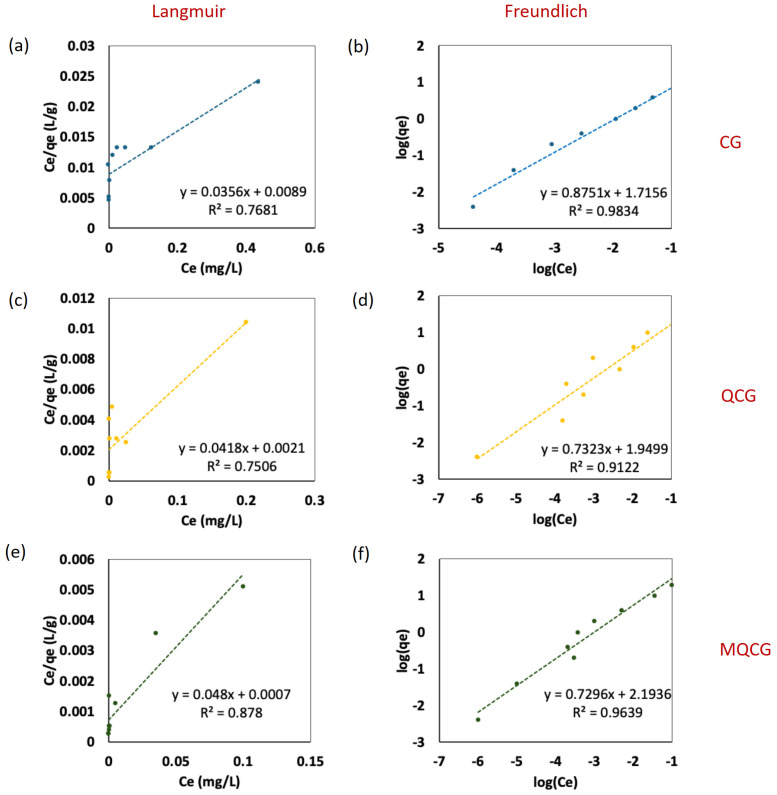
PFOS adsorption isotherm analysis using Langmuir and Freundlich models for CG (**a**,**b**), QCG (**c**,**d**), and MQCG (**e**,**f**). Each data point represents the average of triplicate measurements (standard deviation error bars were omitted for clarity).

An isotherm study was conducted to evaluate the adsorption behavior of the hydrogels for PFOS as a representative of the PFAS. Figure 9a–f presents the adsorption capacities of the hydrogels at equilibrium, which were analyzed using both the Langmuir and the Freundlich isotherm models. The Langmuir model assumes monolayer adsorption on a homogeneous surface with a finite number of identical sites, whereas the Freundlich model describes adsorption on heterogeneous surfaces with sites of varying energies [66]. A comparison of the *R*^2^ values for all samples shows that the adsorption data fit the Freundlich model more accurately (*R*^2^ = 0.98, 0.91, and 0.96) than the Langmuir model (*R*^2^ = 0.77, 0.75, and 0.87), indicating that PFOS adsorption primarily occurs on energetically heterogeneous surfaces and through various interactions [39].

While the energetic heterogeneity in CGs arises from the partial crosslinking of some of the primary amines [63], the quaternization of the QCGs generates heterogeneous adsorption sites by introducing positive charges on some of the amine functional groups [66].

The surface modifications in MQCGs contribute to the formation of more heterogeneous active sites, which also promote multilayer adsorption on the microchannels [67]. Therefore, all three types of hydrogels follow the Freundlich adsorption isotherm model. It is worth noting that the *R*^2^ for the Langmuir model is also relatively high for MQCGs compared to CGs and QCGs. This can be interpreted as a validation of the MQCG’s synergistic design, confirming that a strong, specific electrostatic affinity is combined with underlying structural heterogeneity and a large surface area [68].

The Freundlich isotherm analysis revealed distinct differences in adsorption capacity and intensity across the hydrogel series. The Freundlich adsorption constant (K_F_) progressively increased from 51.91 mg/g for the CG to 89.13 mg/g for the QCG, reaching a maximum of 156.23 mg/g for the MQCG (Figure 9b,d,f). This substantial increase in K_F_ validates the success of both the quaternization and surface area enhancement in increasing the overall PFAS adsorption affinity [69]. Furthermore, the adsorption intensity parameter—*n*, which is an exponent in the Freundlich adsorption isotherm equation—followed a similar trend, increasing from 1.14 in the CG to 1.37 for the QCG and peaking at 1.39 for the MQCG. Since all *n* values are greater than 1, this means that the adsorption process is highly favorable [39]. Higher *n* values for the MQCG confirm the combined effect caused by creating quaternary functional groups and surface channels for enhanced electrostatic interactions and physical entrapment [36].

**Figure 10:**
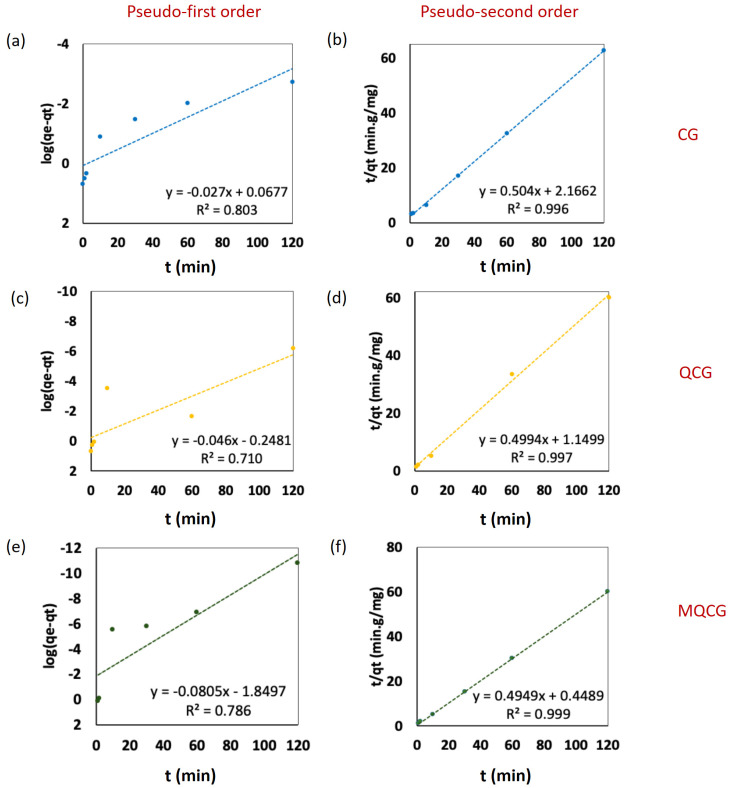
Adsorption kinetics of 500 µg/L PFOS for (**a**) CG, pseudo-first-order model; (**b**) CG, pseudo-second-order model; (**c**) QCGs, pseudo-first-order model; (**d**) QCGs, pseudo-second-order model; (**e**) MQCGs, pseudo-first-order model; and (**f**) MQCGs, pseudo-second-order model. Each data point represents an average of triplicate measurements (standard deviation error bars were omitted for clarity).

To understand the adsorption mechanism and rate, the experimental data were analyzed using both pseudo-first-order and pseudo-second-order kinetic models; while the former assumes physisorption (physical interactions) governed by diffusion, the latter describes chemisorption as the rate-limiting step involving electron sharing or electrostatic interactions [48]. Figure 10a–f demonstrates the PFAS adsorption rates (kinetics of adsorption) for the CGs, QCGs, and MQCGs.

A comparison of the *R*^2^ values of two models for different hydrogels demonstrates a better fitting of all of them with the pseudo-second-order adsorption kinetics (0.80, 0.71, and 0.78 vs. 0.996, 0.997, and 0.999 for CGs, QCGs, and MQCGs, respectively). The CGs have a higher *R*^2^ for pseudo-first-order kinetics compared to QCGs and MQCGs, which reflects that the dominant rate-limiting step in the adsorption process for CGs is the physical adsorption of PFOS rather than chemisorption [48].

The higher *R*^2^ for the pseudo-second-order kinetics of MQCGs demonstrates that the rate is controlled by chemisorption, meaning that the surface chemical reaction—the electrostatic binding between the positively charged quaternary ammonium sites (N+) and the PFAS anions—is the dominant step. This result provides evidence for the successful introduction of the specific, high-energy electrostatic binding sites via quaternization, which dictates the overall reaction rate.

All three hydrogels demonstrate a relatively comparable equilibrium adsorption capacity for PFOS. However, the adsorption rate constant (*K*_2_) is significantly increased by the quaternization followed by the surface modification. *K*_2_ values increased from 0.117 g/mg·min in CGs to 0.217 g/mg·min in QCGs and reached 0.545 g/mg·min in MQCGs.

*K*_2_ values represent the pseudo-second-order rate constant, indicating the rate of chemisorption between PFAS molecules and hydrogel surface functional groups. A higher *K*_2_ value reflects a faster adsorption process [70]. This remarkable enhancement in the kinetic rate suggests that the quaternization and surface modifications, particularly in MQCGs, significantly improved the availability and accessibility of active sites, allowing for simultaneous hydrophobic, electrostatic, and physical interactions and, hence, a faster PFAS uptake [43,67].

Summarily, the adsorption behavior of the MQCGs, fitted to the Freundlich isotherm model, and the pseudo-second-order kinetics indicate that the PFOS adsorption is dominated by the chemisorption (electrostatic) that occurs on a heterogeneous surface (increased surface area) with multilayer adsorption characteristics [48,67,70].

Table 2 summarizes the isotherm and kinetics modeling parameters for the hydrogels’ adsorption of PFOS. It should be noted that the data points corresponding to complete PFOS removal ($C_{e}$ < detection limit) in both the isotherm and kinetics analysis were excluded from the model fitting due to the possibility of reaching the instrument detection limit; however, they are shown on the plots as reference points representing the maximum theoretical uptake.

#### 2.2.3. The Effect of pH on PFAS Removal Efficiency

**Figure 11:**
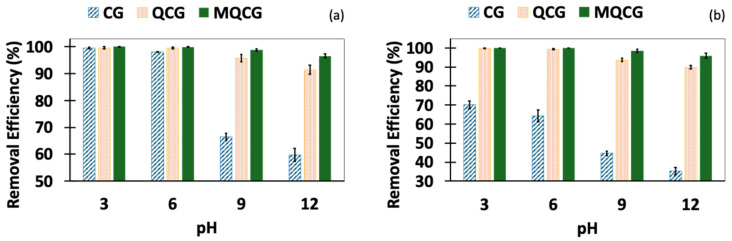
The effect water pH on the adsorption of (**a**) PFOS and (**b**) PFBS. (Error bars represent the standard deviation of at least three replicates for each data point).

Figure 11 illustrates the effect of the water pH on the adsorption of long-chain (PFOS) and short-chain (PFBS) PFAS. The removal efficiencies are shown in pH levels ranging from 3 to 12. Figure 11a illustrates that all hydrogel types exhibit significantly enhanced PFOS adsorption under acidic conditions (pH = 3), which can be attributed to the effective protonation of primary amine groups at lower pH levels [54].

However, the QCG maintains a strong adsorption performance, with <95% at pH 9 and <90% at pH 12. This performance is improved in MQCGs, which exhibit ~98% adsorption at pH 9 and ~96% at pH 12. This enhancement is likely due to the combined effects of strengthened hydrophobic and electrostatic interactions, as well as physical interactions [40].

A similar trend is observed for the adsorption of the short-chain PFAS, PFBS, across different pH levels (Figure 11b). All hydrogels exhibit a higher PFBS removal efficiency at an acidic pH than at a neutral pH. The CG demonstrates significantly lower PFBS adsorption than PFOS, highlighting its limited interaction (mostly hydrophobic interaction) with short-chain PFAS [71]. In contrast, the QCG maintains high PFBS adsorption (~90%) even at pH 12 and performs well across the entire pH range tested (3, 6, 9, and 12). Notably, the MQCG exhibits superior PFBS removal, achieving 100% adsorption at pH 3 and 6 and maintaining >95% efficiency at pH 9 and 12.

These results obtained at different pH values indicate that the primary mechanism of PFAS adsorption for QCGs and MQCGs is strong electrostatic attraction. Quaternization introduces permanent positive charges to the QCGs and MQCGs (Figure 1), which strongly attract the anionic PFAS head groups. This is confirmed by the high, stable removal efficiency observed across the broad pH range of 3 to 12. This pH-independent performance demonstrates that adsorption relies on fixed quaternary ammonium charges, ensuring a robust Coulombic interaction regardless of the water pH.

#### 2.2.4. Regeneration Studies

**Figure 12:**
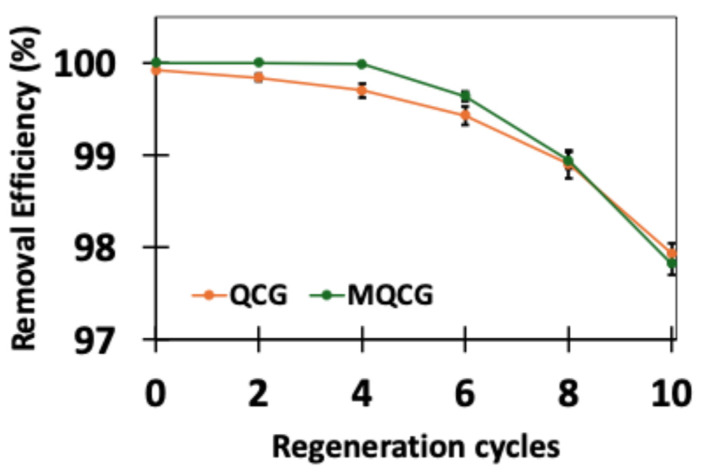
PFOS removal efficiency for different regeneration cycles of QCGs vs. MQCGs. (Error bars represent the standard deviation of at least three replicates for each data point).

Reusing hydrogels contributes to long-term cost-effectiveness and environmental sustainability. Figure 12 illustrates the PFOS removal efficiency of the hydrogels after multiple regeneration cycles. The desorption of PFAS using salt solutions was not effective for the CGs; therefore, they were excluded from the regeneration studies.

As seen in Figure 12, both the QCG and MQCG are reusable for up to 10 regeneration cycles. MQCGs maintain higher removal efficiency during the first eight cycles. However, QCGs slightly surpass MQCGs after eight cycles. This decrease is due to the presence of the microchannels on the surface of MQCGs, which are better able to entrap PFAS due to their large surface area. Conversely, the large surface area of the microchannels also makes later desorption more difficult. Nonetheless, the difference in efficiency at these later stages remains minimal: 98% for QCGs vs. 97.35% for MQCGs.

The larger standard deviation values found for both hydrogels with further regeneration cycles reflect cumulative experimental differences across repeated adsorption/desorption steps. The data nevertheless demonstrate that MQCGs retain a high adsorption capacity across multiple cycles [39].

Notably, achieving such high removal efficiencies using a simple NaCl solution provides evidence that the underlying adsorption mechanism is that of anion exchange. This mechanism is driven by the competitive displacement of the PFAS by chloride ions (Cl−). Using such a low-cost, readily available salt solution rather than energy-intensive thermal or corrosive solvent methods is an advantage of the MQCGs that supports their long-term sustainability and economic viability for potential real-world water treatment applications.

Regeneration, while cost-effective, transfers adsorbed PFAS to a concentrated liquid waste stream (eluent). This stream necessitates the final destructive treatment for complete environmental remediation. Concentrating the PFAS as a small volume significantly facilitates advanced destruction technologies (e.g., electrochemical oxidation or super critical water oxidation) compared to treating the original diluted water source [72].

#### 2.2.5. PFAS Removal Efficiency in Simulated Tap Water

**Figure 13:**
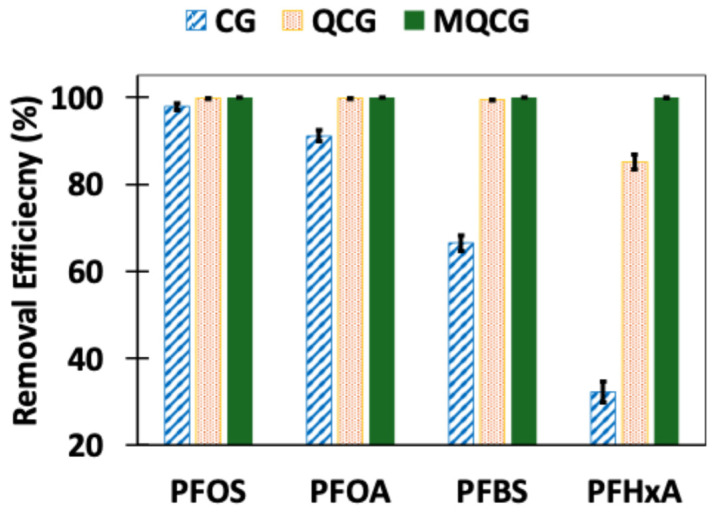
Removal efficiency of hydrogels for different PFAS in tap water. (Error bars represent the standard deviation of at least three replicates for each data point.).

Long-chain and short-chain PFAS adsorption in simulated tap water (including MgSO_4_ and NaCl co-ion contaminants) was investigated in relation to the real-world application of chitosan hydrogels. Figure 13 illustrates that the removal efficiency decreased slightly for QCGs in tap water compared to DI water solutions. The removal efficiencies of QCGs are 99.74, 99.7, 99.42, and 85.14% for PFOS, PFOA, PFBS, and PFHxA, respectively. The reduction in the short-chain PFAS removal for QCGs may be due to the co-ions blocking or saturating some of the positively charged amine groups, leading to the reduced removal efficiency of the QCGs [39,54].

The removal efficiency of MQCGs was not significantly affected by the presence of co-ions due to the fast adsorption of PFAS (as indicated by high *K*_2_ values from the kinetic studies) and the employment of multiple adsorption mechanisms, including electrostatic, hydrophobic, and physical entrapment [61]. According to the kinetic and isotherm studies (*K*_2_ = 0.545 mg·g^−1^·min^−1^ and K_F_ = 156.23 mg·g^−1^), approximately 98% of the short- and long-chain PFAS mixture was removed from tap water within 30 min. This represents a significant improvement in both the adsorption capacity and rate compared to previously reported chitosan hydrogels, which achieved ~97% removal of only PFOA from deionized water within 1 h under contaminant-free conditions [73].

These experimental results provide evidence that the surface modification of quaternized chitosan hydrogels has effectively increased the adsorption active sites, leading to an increase in the adsorption rate, capacity, and efficient PFAS removal in the presence of co-contaminants in tap water.

MQCGs have a larger surface area compared to QCGs due to their surface microchannels, as confirmed by the BET analysis (Figure 7). This increased surface area exposes more primary amine groups, resulting in a higher positive charge after quaternization, as illustrated by zeta potential measurements (Figure 5). The enhanced positive charge strengthens electrostatic interactions with the negatively charged PFAS headgroups. The greater hydrophobic surface area improves hydrophobic interactions with PFAS chains [73].

**Figure 14:**
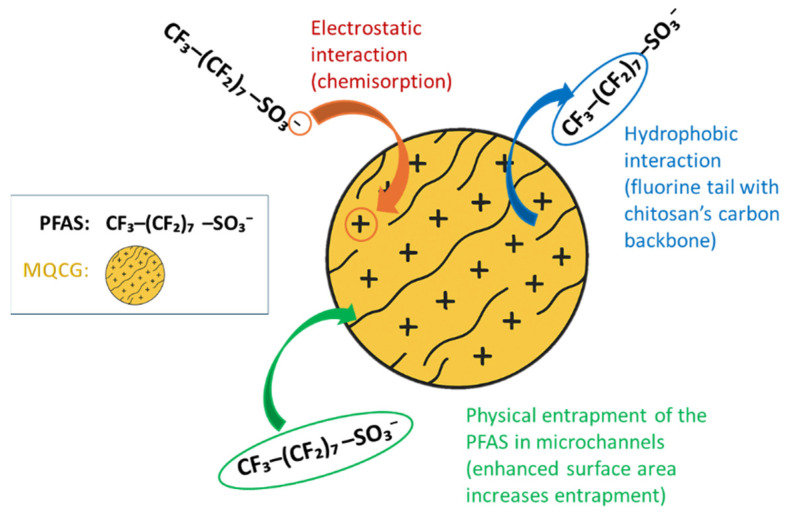
Schematic diagram of the proposed adsorption mechanisms between MQCGs and PFAS.

In addition, the surface microchannels promote physical interactions, such as van der Waals contacts along the channel walls, which assist in capturing both long- and short-chain PFAS. Together, these effects account for the superior adsorption performance of MQCGs compared to QCGs [74]. Figure 14 illustrates the proposed adsorption mechanisms of PFAS on the MQCGs.

## 3. Conclusions

Novel chitosan-based hydrogels were developed for the adsorption of both long-chain and short-chain PFAS. Genipin, a bio-based crosslinker, was used to enhance the hydrogels’ stability in water, while GTMAC was incorporated to introduce permanent positive charges on the hydrogel surface, reducing pH sensitivity and increasing electrostatic interactions with negatively charged PFAS. Additionally, blending chitosan with PEG-6000, followed by solvent extraction, generated microscale surface channels on the hydrogels, increasing the surface area and enhancing PFAS adsorption through hydrophobic interactions, electrostatic attraction, and physical interactions.

The hydrogels’ adsorption capacities and ability to remove a range of PFAS compounds were evaluated. Isotherm studies revealed that the adsorption mechanism followed the Freundlich model, indicating heterogeneous active sites, with MQCGs demonstrating the highest Freundlich adsorption constant among all hydrogels (*K*_F_ = 156.23 mg/g). Kinetic studies showed that adsorption followed a pseudo-second-order mechanism, with MQCGs exhibiting the highest adsorption rates among the hydrogel types (*K*_2_ = 0.545 mg·g^−1^.min^−1^).

MQCGs effectively removed PFAS across a wide pH range (3–12) and were successfully regenerated for up to 10 cycles using a simple method, maintaining a high removal efficiency < ~98%. The hydrogels also demonstrated the efficient removal of PFOS, PFOA, PFBS, and PFHxA from simulated tap water samples containing co-ion contaminants, such as MgSO_4_ and NaCl, in the same batch.

A comparative assessment with recently published studies of chitosan-based adsorbents (Table 3) highlights the characteristics of the MQCGs and their advantages. While PEI-grafted beads and aerogel-based materials can achieve high adsorption capacities, they often exhibit slower kinetics for short-chain PFAS, limited pH stability, or rely on toxic crosslinkers such as epichlorohydrin or glutaraldehyde [28,37,40,74]. In contrast, MQCG achieves rapid PFAS removal (mixture of short and long-chain PFAS > 98% within 30 min) over a wide pH range (3–12) via a non-chlorinated synthesis route while maintaining performance in simulated tap water.

This proof-of-concept study demonstrates the highly efficient removal of PFAS from water using a novel bio-based hydrogel. Moreover, this study presents a sustainable approach for potential water treatment applications, offering rapid, highly efficient removal of both long-chain and short-chain PFAS using reusable, bio-based hydrogels under environmentally relevant concentrations. Notably, our approach avoids the use of nanoparticles and eliminates the need for the incineration of adsorbents after a single use.

Future work will focus on optimizing the hydrogels to enhance their equilibrium adsorption capacity and explore their potential for real-world groundwater and well water treatment by conducting dynamic (column) experiments. Additionally, the biofouling resistance of these chitosan hydrogels will be evaluated under long-term use in tap water or groundwater environments.

## 4. Materials and Methods

### 4.1. Materials

Chitosan powder (MW: 100–150 KDa; purity: 98%; CAS No.: 9012-76-4; Product No.: B-95-545381) was obtained from Chitolytic company (Toronto, ON, Canada). Key specifications include a high degree of deacetylation (DDA) of 95.6% and a viscosity of 66 mPas, measured in a 1% *w*/*v* solution in acetic acid at 25 °C. Polyethylene glycol (MW = 1000, 3000, and 6000 g/mole), genipin, glycidyl trimethyl ammonium chloride (GTMAC), sodium hydroxide (NaOH), glacial acetic acid, and ethanol were purchased from Fisher Scientific (Pittsburgh, PA, USA) and were used without further purification. The PFAS chemicals, PFOS (Perfluorooctanesulfonate), PFOA (Perfluorooctanoic acid), PFBS (Perfluorobutanesulfonate), and PFHxA (Perfluorohexanoic acid), used in adsorption experiments were provided by Transene Company (Danvers, MA, USA) (see Table S1 in the Supporting Information). PFAS standards were from Accustandards (New Haven, CT, USA).

### 4.2. Synthesis and Surface Modification of Chitosan Hydrogels

#### 4.2.1. Synthesis of Chitosan Hydrogels

A 3 wt.% chitosan solution was prepared by dissolving 3 g of chitosan in 97 mL of a 2 *v*/*v*% glacial acetic acid–water mixture and stirring it overnight at room temperature. The resulting viscous solution was then used to generate chitosan hydrogels using a 20 μL pipette. Uniform droplets of the chitosan solution were dripped into a 1 M NaOH solution and left at room temperature for 2 h to form spherical gels [51].

#### 4.2.2. Crosslinking of Chitosan Hydrogels

The hydrogels were washed multiple times with DI water until a neutral pH was reached. Then, a 2.5 wt.% genipin solution (1.5 mL/g of hydrogels) was added, and the batch was left for 24 h to allow crosslinking. Afterward, the crosslinked hydrogels were thoroughly washed with DI water to remove any unreacted genipin [44].

#### 4.2.3. Surface Modification of Crosslinked Quaternized Chitosan Hydrogels

To enhance the surface area as well as the accessibility of the positive charges on chitosan hydrogels, modified hydrogels with microchannels on the surface were created using “sacrificial templating” of PEG-6000 [75]. For this purpose, PEG-6000 and chitosan were blended in a 2 *v*/*v*% glacial acetic acid–water mixture and stirred at room temperature using an overhead mechanical stirrer. The blend was then added dropwise into a 1 M NaOH solution using a 20 µL pipette and left at room temperature for 2 h to form spherical gels.

The hydrogels were crosslinked following the same procedure as the chitosan hydrogels. Then, they were washed multiple times with an ethanol/water mixture before undergoing quaternization. Due to the higher mobility of PEG-6000 (lower MW compared to chitosan) and slow crosslinking of chitosan, PEG-6000 molecules tend to migrate to the surface of the hydrogels. Following that, the selective solubilization of PEG in an ethanol/water mixture leads to the formation of microchannels on the surface of the hydrogels (see Sections S2 and S3 in the Supporting Information, Table S2, and Figures S1–S3) [76]. MQCG1 and MQCG2 refer to surface-modified quaternized chitosan hydrogels prepared with 1 wt.% and 2 wt.% PEG-6000, respectively. Unless otherwise specified, the term MQCG (without a numeric label) refers to MQCG1 throughout this work.

#### 4.2.4. Quaternization of Chitosan Hydrogels

The crosslinked chitosan hydrogels were immersed in a 20 wt.% solution of glycidyl trimethyl ammonium chloride (GTMAC) and left for 24 h to complete the quaternization process. The quaternized chitosan hydrogels were washed with DI water and dried overnight at 80 °C. The resulting positive charge on the surface of the hydrogels was then evaluated by zeta potential measurements using a dynamic light scattering instrument.

### 4.3. Characterization of Chitosan Hydrogels

#### 4.3.1. Fourier Transform Infrared–Attenuated Total Reflectance Spectroscopy

Fourier Transform Infrared (FTIR) spectroscopy analysis was conducted using a Nicolet iS50 instrument (Thermo-Scientific, Waltham, MA, USA) in attenuated total reflectance (ATR) mode, equipped with a diamond crystal. The instrument analyzed the crosslinking and quaternization of hydrogels across the range of 4000–400 cm^−1^ by monitoring the conversion of the primary to secondary amines in both reactions. Each spectrum resulted from 128 scans at a resolution of 4 cm^−1^.

#### 4.3.2. Microscopy and Elemental Analysis

Optical microscopy (OM) (Zeiss V20 stereomicroscope, Tokyo, Japan) was used to capture three-dimensional images of the hydrogels. A field-emission Scanning Electron Microscope (FE-SEM) JEOL JSM 7401F (Tokyo, Japan) was used to observe the surface morphology of the original (CG), quaternized (QCG), and surface-modified quaternized (MQCG) chitosan hydrogels. The images were captured using 5 kV accelerated voltage and in low- and high-magnification (OM and SEM) modes. To avoid charging effects, hydrogels were gold sputtered for SEM imaging and carbon coated for elemental mapping via Electron Dispersive Spectroscopy (EDS) analysis, using a Denton Vacuum Desk IV sputter (Moorestown, NJ, USA).

#### 4.3.3. Thermogravimetric Analysis

To investigate the effect of crosslinking, quaternization, and surface modification on thermal degradation of the hydrogels, thermogravimetric analysis was performed using a TGA5500 discovery model from TA Instruments (Newcastle, DE, USA). The temperature range was from 50 °C to 800 °C, and the ramp heating rate was 5 °C/min under N_2_ gas flow.

#### 4.3.4. Surface Area Measurement

A Micromeritics ASAP2020 Plus Automatic Micropore and Chemisorption Analyzer (Norcross, GA, USA) was used to measure the surface area (BET and *t*-plot) of CGs, QCGs, and MQCGs. The surface area measurement is critical for understanding the effect of increasing the contact area between the hydrogels and PFAS on PFAS uptake. The Brunauer–Emmett–Teller (BET) method calculates the total surface area in the relative pressure range of 0.05–0.30 P/P_0_. To further differentiate between external surface area and micropore contributions, the *t*-plot method was applied using a standard thickness curve based on reference materials.

#### 4.3.5. Zeta Potential Measurements

A Horiba SZ-100 dynamic light scattering instrument (Kyoto, Japan) was used to evaluate the surface charge of the hydrogels after quaternization. The surface charge of the hydrogels was measured in the range of −200 to +200 mV, and each measurement was repeated 5 times. The DI water used to disperse the hydrogels had a pH of 6.5.

### 4.4. Quantification of PFAS via Liquid Chromatography/Mass Spectroscopy Analysis

PFAS adsorption analysis was conducted using a Waters Xevo G2-XS UPLC-QTOF mass spectrometer (Milford, MA, USA) at the University of Massachusetts Lowell Core Research Facilities (CRF). To ensure measurements remained well above the instrument’s detection and sensitivity limits, PFAS concentrations in all water samples were maintained at or above 1 µg/L throughout the analyses. An Acquity BEH C18 column was used to separate analytes with a mobile phase composed of ammonium acetate aqueous solution and methanol. Commercial PFAS standards diluted to various concentrations were used to calibrate the Liquid Chromatography/Mass Spectroscopy (LC/MS) system. The initial and final PFAS concentrations (*C*_i_ and *C*_f_) were measured and used to calculate removal efficiency (R%) and adsorption capacity (*q_e_*, mg/g) of the bio-adsorbents. All measurements were performed in triplicate, with averages and standard deviations reported. The DI water used had a pH of 6.5.

### 4.5. Equilibrium PFAS Removal Efficiency and Adsorption Capacity

The adsorption experiments for CGs, QCGs, and MQCGs were conducted using PFAS-spiked DI water. All adsorption measurements were conducted in triplicate, and the averages were plotted with error bars denoting the standard deviations. The experiments were performed in polypropylene centrifuge tubes to minimize PFAS sorption to secondary surfaces [77]. Additional experiments were carried out to investigate and optimize the conditions affecting PFAS removal efficiency. These included contact time, the impact of quaternization on the pH sensitivity of the hydrogels, initial hydrogel concentration, regenerability of the hydrogels, and the removal efficiency in simulated tap water.

The removal efficiency (%) was calculated using the following equation:

(1) $$R\left(\%\right)=((C_{i}-C_{f})/C_{i})\times 100\%$$

$C_{i}$ is the initial concentration of PFAS (mgL), and $C_{f}$ is the final concentration of PFAS (mgL) in the solution after adsorption was measured using Q-TOF LC/MS.

The adsorption capacity $q_{e}$ (mgg) of the PFAS compounds at equilibrium (24 h of adsorption of a 500 µg/L PFAS solution) was determined using

(2) $$q_{e}=\left(\left(C_{i}-C_{f}\right)\times V\right)/m$$

$V\;\left(L\right)$ is the volume of the PFAS solution, and m (g) is the mass of the adsorbent used [36,77].

### 4.6. Isotherm and Kinetics of Adsorption of PFAS on Hydrogels

To evaluate the adsorption behavior at different PFAS concentrations, the isotherms of the hydrogels were studied using the Langmuir and the Freundlich models [66].

For the isotherm analysis, the amount of PFAS removed by each hydrogel (CG, QCG, and MQCG) was investigated at different starting concentrations once the system had reached equilibrium. In our study, equilibrium is defined as 24 h, so time does not change in this part of the analysis—it is fixed. The amount of PFAS adsorption across a range of initial concentrations was compared for each type of hydrogel (CG, QCG, and MQCG) over 24 h. These values were then plotted and fitted to the Langmuir or the Freundlich isotherm models.

Specifically, PFAS solutions with concentrations ranging from 1 to 5000 μg/L were used to assess the impact of initial PFAS concentration on the hydrogels’ adsorption capacity at fixed adsorbent mass and contaminant solution volume. The selected concentration range ensured detection above the Q-TOF LC/MS instrument’s minimum sensitivity threshold while encompassing a broad span necessary for accurate isotherm analysis. The adsorption equilibrium time was set to 24 h. Removal efficiencies of the different hydrogels at equilibrium were used to calculate adsorption capacities, and the results were curve-fitted to the Langmuir and the Freundlich isotherm models according to the equations described in Section S4 of the Supporting Information.

All PFAS adsorption measurements in the LC/MS analysis were performed in triplicate for each type of hydrogel, at concentrations ranging from 1 to 5000 μg/L, and each point on the isotherm curves represents the average of those three measurements.

The kinetics of adsorption were evaluated using pseudo-first- and pseudo-second-order models to study the adsorption rate [48]. The setup for these experiments was different than the isotherm experiments. Here, the initial PFAS concentration stayed the same (500 µg/L), and instead, the rate of PFAS removal for each type of hydrogel (CG, QCG, and MQCG) was monitored over time, ranging from 0 to 1440 min (48 h). The hydrogels were placed in a 40 mL solution of 500 µg/L PFAS on a rotary mixer at 40 rpm. Samples were then collected at different times, starting at 1 min. The concentrations of PFAS at each time point were measured in triplicate using LC/MS.

However, PFAS concentrations measured from only the first 120 min were used to calculate the adsorption capacity at different time intervals. Only these calculated adsorption capacity values were used to fit the pseudo-first-order and pseudo-second-order kinetic models. This is because the kinetic models are more reliable at early time points (see Section S4 in the Supporting Information).

The models were fitted to the mean values of removal efficiency obtained from at least three independent measurements from the LC/MS-QTOF analysis, and the associated standard deviations are reported in Table 1 and Table 2 but omitted in Figure 9 and Figure 10.

### 4.7. The Effect of the Water pH on the Adsorption Efficiency of the Hydrogels

To evaluate the impact of quaternization and surface modification on adsorption efficiency, CGs, QCGs, and MQCGs were each dispersed in 40 mL of 500 µg/L PFAS solutions across a pH range of 3 to 12. The removal efficiency and adsorption capacities were calculated and compared for each sample.

### 4.8. The Regenerability of the Hydrogels

The adsorbents were regenerated using a 0.025 M NaCl solution. The hydrogels were dispersed in the salt solution for approximately an hour to facilitate desorption. Then they were recollected and washed with DI water. This process was repeated for up to 10 cycles of adsorption and desorption. The efficiency of PFAS adsorption was evaluated using the same procedure used for the pH effect experiment.

### 4.9. The Removal Efficiency of PFAS from Simulated Tap Water

The removal efficiency and adsorption capacity of the hydrogels were assessed using simulated tap water containing 5 mM NaCl and 0.5 mM MgSO_4_ as co-contaminant ions, spiked with 20 µg/L of each PFAS compound (PFOA, PFOS, PFHxA, and PFBS) in a mixed solution. The simulated tap water was prepared following the procedures from previous relevant studies [78]. The adsorption analysis and removal efficiency followed the same procedure described for the previous steps.

## Figures and Tables

**Table 1:** Removal efficiency and adsorption capacity of hydrogels for 500 µg/L PFAS at equilibrium.

PFAS	Removal Efficiency (%)			Adsorption Capacity *q_e_* (mg g^−1^)		
	CG	QCG	MQCG	CG	QCG	MQCG
PFOS	98.12 ± 0.51	99.84 ± 0.03	100 ± <0.01	1.962 ± 0.031	1.997 ± 0.010	≥2.000 ± <0.001 *
PFOA	92.18 ± 1.20	99.48 ± 0.11	100 ± <0.01	1.840 ± 0.090	1.980 ± 0.011	≥2.000 ± <0.001 *
PFBS	64.43 ± 2.11	98.70 ± 0.40	99.98 ± 0.01	1.291 ± 0.120	1.974 ± 0.020	≥1.999 ± <0.001 *
PFHxA	34.32 ± 3.04	94.30 ± 0.53	99.96 ± 0.03	0.691 ± 0.100	1.884 ± 0.041	≥1.998 ± 0.001 *

* C_e_ < instrument detection limit; reported values represent minimum adsorption capacities under test conditions. The standard deviation values for triplicate measurements are reported.

**Table 2:** Isotherm and kinetic modeling parameters for the adsorption of the PFOS by hydrogels.

	Freundlich Isotherm Parameters *			Pseudo-Second-Order Kinetics Parameters *		
Hydrogel	*R* ^2^	Freundlich Capacity Constant, K_F_ (mg/g)	Adsorption Intensity, *n*	*R* ^2^	Equilibrium Adsorption Capacity, 500 ppb PFOS, *q_e_* (mg/g)	Adsorption Rate Constant, *K*_2_ (mg·g^−1^·min^−1^)
CG	0.981	51.91 ± 1.49	1.14 ± 0.02	0.996	1.980 ± 0.003	0.117 ± 0.001
QCG	0.910	89.13 ± 1.89	1.37 ± 0.01	0.997	1.990 ± 0.002	0.217 ± 0.002
MQCG	0.960	156.23 ± 1.71	1.39 ± 0.01	0.999	2.020 ± <0.001	0.545 ± 0.001

* The standard deviation values for triplicate measurements are reported.

**Table 3:** A comparison of MQCGs with recently published studies of chitosan-based PFAS adsorbents.

Adsorbent Material	PFAS Type	Time to Remove > 98% PFAS	pH & Test Condition	Synthesis Procedure	Ref.
Surface-Modified Quaternized Chitosan Hydrogels (MQCG)	PFOS, PFOA PFBS, PFHxA	30 min (short- and long-chain PFAS mixture)	pH 3–12, Simulated tap water with co-ion contaminants	Facile synthesis, no toxic chlorinated crosslinkers, facile regeneration, maintained performance in the presence of co-ions	This study
PEI-grafted Chitosan Beads (GCBs)	PFOS, PFOA, PFBS, PFBA	5 min (long-chain), 3 h (short chain)	Optimal at pH 6; performance drops at pH > 11	Complex synthesis using epichlorohydrin (carcinogenic), reduced performance in real water, slow short-chain PFAS adsorption	[28]
Glutaraldehyde-Chitosan-Polyethyleneimine Aerogel	Mixture of 12 PFAS	24 h	pH 2–10	Uses glutaraldehyde (toxic crosslinker); very slow kinetics limit practical flow-through applications	[40]
Chitosan-coated F-COF	PFOS, PFOA, GenX	4 h	Neutral pH	Using *o*-dichlorobenzene (toxic solvent); difficult to regenerate, slow kinetics	[37]
Magnetic Chitosan Spheres (Fe_3_O_4_)	PFOS, PFOA	60 min	pH 4–10	Uses glutaraldehyde (toxic crosslinker); only tested for long-chain PFAS removal, slow kinetics	[74]

## Data Availability

The data presented in this study are available on request from the corresponding author.

## Supplementary Materials

The following supporting information can be downloaded at https://www.mdpi.com/article/10.3390/gels12010014/s1. Table S1. PFAS chemicals, their molecular structure, and their counterions. Table S2. PEGs used as sacrificial templating agents. Figure S1. Optical microscopy images of the hydrogels’ surfaces with PEG as sacrificial agent. Figure S2. SEM images of MQCGs with (a) 1 wt.%, (b) 2 wt.%, and (c) 3 wt.% of PEG6000. Equations used in the isotherm and kinetics of adsorption studies. Figure S3. Cross-sectional SEM images of the hydrogels at two magnifications: (a,b) CG at ×2000 and ×10,000, respectively; (c,d) QCG at ×2000 and ×10,000; (e,f) MQCG1 at ×2000 and ×10,000; (g,h) MQCG2 at ×2000 and ×10,000. Figure S4. Raw data plots of the zeta potential of the hydrogels obtained by DLS analysis [25,38,67,75,79,80,81].
