# Supplementary material for: Highly Efficient Removal of PFAS from Water Using Surface-Modified Regenerable Quaternized Chitosan Hydrogels
Source: Gels. 2025 Dec 24;12(1):14. doi: 10.3390/gels12010014 (PMC12841056; doi:10.3390/gels12010014)
Supplement: Supplementary file 1 [file gels-12-00014-s001.zip › Supporting Information_Gels-4015424.pdf]

Supporting Information

Highly Efficient Removal of PFAS from Water using Surface-Modified Regenerable Quaternized Chitosan Hydrogels

Mohammad Bagheri Kashani,[1] *Lingfei Fan,*[2] *Weile Yan,*[2] *Bridgette M. Budhlall,*[1,] *

[1] Department of Plastics Engineering, University of Massachusetts Lowell, MA, 01854, USA.

[2] Department of Civil and Environmental Engineering, University of Massachusetts, Lowell, MA, 01854, USA.

Corresponding Author

* Bridgette M. Budhlall, Email: Bridgette_Budhlall@uml.edu

1. PFAS used in the study

Table S1 represents the PFAS chemicals, their molecular structure, and their counterions.

Table S1. PFAS chemicals used in the adsorption tests

Chemical	Molecular Structure	Functional Group	Typical Counterion	Carbon Chain Length
PFOS (Perfluorooctanesulfonate)	**CF_3–$(CF_2)_7$–SO_3^-**	Sulfonate ($-SO_3^-$)	K^+, NH_4^+, or Na^+	C8 (Long chain)
PFOA (Perfluorooctanoic acid)	**CF_3–$(CF_2)_6$–COO^-**	Carboxylate ($-COO^-$)	NH_4^+, Na^+, or K^+	C8 (Long chain)
PFBS (Perfluorobutanesulfonate)	**CF_3–$(CF_2)_3$–SO_3^-**	Sulfonate ($-SO_3^-$)	K^+, NH_4^+, or Na^+	C4 (Short chain)
PFHxA (Perfluorohexanoic acid)	**CF_3–$(CF_2)_4$–COO^-**	Carboxylate ($-COO^-$)	Na^+ or NH_4^+	C6 (Short chain)

2. Surface microchannels created via sacrificial templating of PEG

To enhance PFAS interaction via electrostatic, hydrophobic, and physical mechanisms, surface channels were formed on the hydrogels. Surface micro-sized channels are preferred over larger channels or pores, as the latter may trap PFAS within the hydrogels and hinder their desorption.[1] The facile desorption is crucial for the regeneration of the hydrogels. These surface channels were prepared using sacrificial templating of PEG.[2]

For this purpose, chitosan was initially dissolved in a 2 v/v% glacial acetic acid/water solution. Then, PEGs with different molecular weights and different weight percentages were added to the solution and dissolved using an overhead stirrer. **Table S2** represents various PEGs used to generate surface structures and the surface texture created.

Table S2: PEGs used as sacrificial templating agents for surface microchannels creation

PEG MW	Wt.%		Notes
	1	2	
PEG-1000	Not much different than neat chitosan	Surface holes and not channels	Not preferred for PFAS adsorption
PEG-3000	Shallow Surface holes and not channels	Deep surface holes, with surface channels at some locations	Not suitable for regeneration
PEG-6000	Shallow Surface channels, Acceptable mechanical stability	Deep surface channels and poor hydrogels' mechanical properties	1 wt.% with desired properties

Figure S1 presents optical microscopy images of the hydrogel surfaces, highlighting the surface structures formed using PEG as a sacrificial agent. As the molecular weight of PEG increases, the morphology shifts from isolated holes to well-defined channels. When 1 wt.% PEG-6000 is used, the uniform surface channels are observed.

However, increasing the concentration to 2 wt.% results in deeper channels, which compromises the mechanical stability of the hydrogel. Samples containing surface holes or pores

(PEG-1000 and PEG-3000) are not ideal for applications requiring regenerability, as previously discussed.

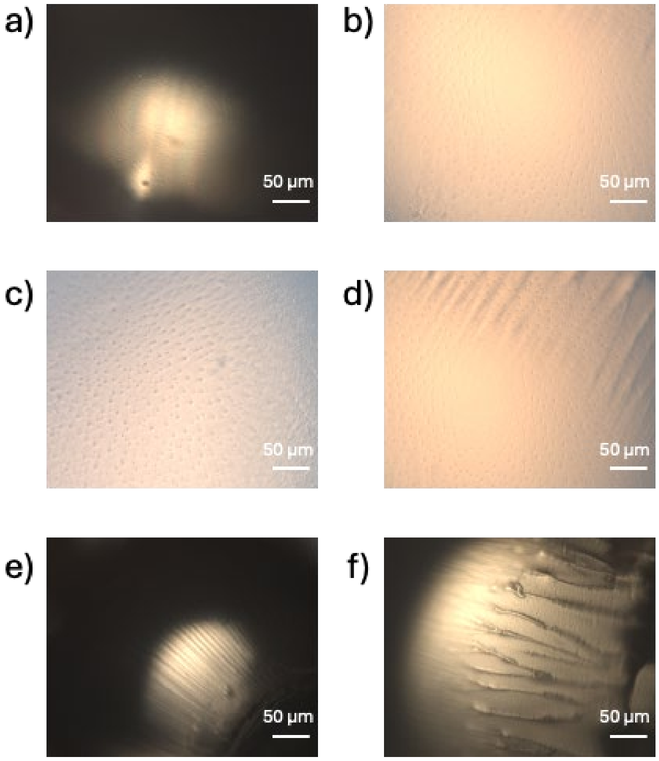

Figure S1: The optical microscopy images of the hydrogels' surfaces with PEG as sacrificial agent: a) 1 wt.% PEG-1000, b) 2 wt.% PEG-1000, c) 1 wt.% PEG-3000, d) 2 wt.% PEG-3000, e) 1 wt.% PEG-6000, and f) 2 wt.% PEG-6000.

It should be noted that high weight ratios of the sacrificial agent (PEG) may alter the inherent properties of the hydrogels. PEG can also form hydrogen bonds with chitosan, making its removal complicated and time-consuming. Therefore, the weight ratios of PEGs, regardless of molecular weight, did not exceed 2 wt.% in this study.[2] **Figure S2** shows the SEM images of MQCGs prepared with 1, 2, and 3 wt.% PEG6000 as a sacrificial templating agent. Hydrogels containing 2 and 3 wt.% PEG6000 exhibited poor structural integrity and fractured easily during routine handling and weighing, specifically for regeneration studies. In contrast, MQCGs prepared with 1 wt.% PEG6000 retained their morphology after the sacrificial templating process.

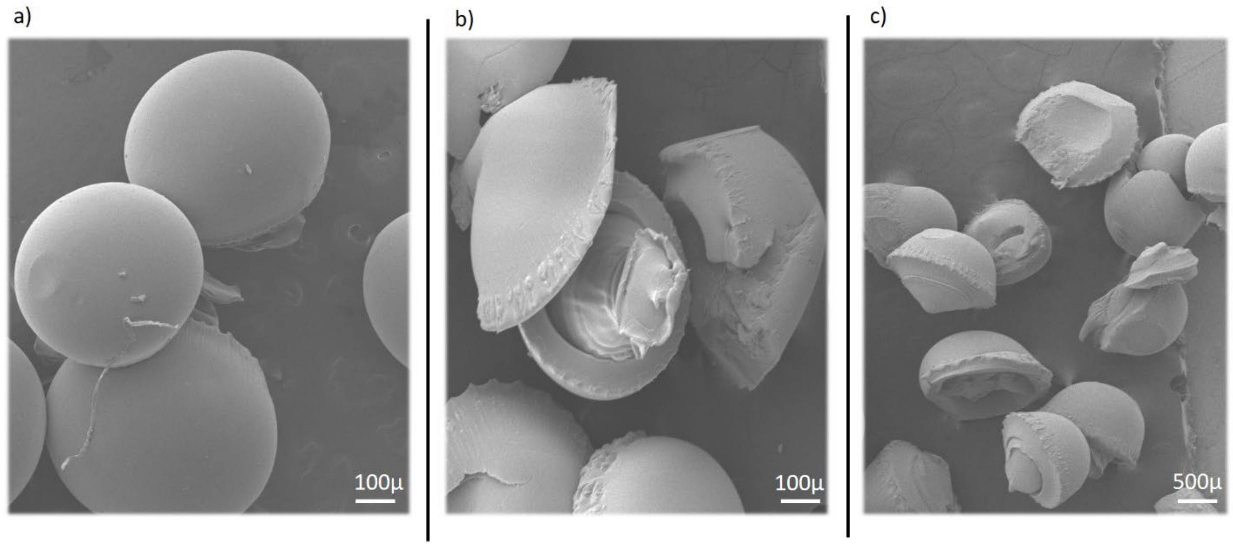

Figure S2: The scanning electron microscopy images of MQCGs with a) 1 wt.%, b) 2 wt.%, and c) 3wt.% of PEG6000

3. **Cross-sectional imaging of hydrogels (internal porosity comparison)**

Cross-sectional imaging of the hydrogels (CG, QCG, MQCG1, and MQCG2) was conducted using scanning electron microscopy. The hydrogel samples were dehydrated through a graded ethanol series (30%, 50%, 70%, and 90% v/v), followed by three successive immersions in absolute ethanol (100%), with each step maintained for 10 min to ensure complete solvent exchange.[3] The ethanol-saturated samples were immediately transferred to a critical point dryer (Tousimis Samdri-795, MD, USA), where ethanol was replaced with liquid carbon dioxide (CO_2) via multiple soak–purge cycles. Subsequently, the system was heated above the critical point of carbon dioxide (31 °C, 73.8 bar), and the chamber was slowly depressurized under controlled conditions to preserve the internal pore structure and prevent pore collapse.[3]

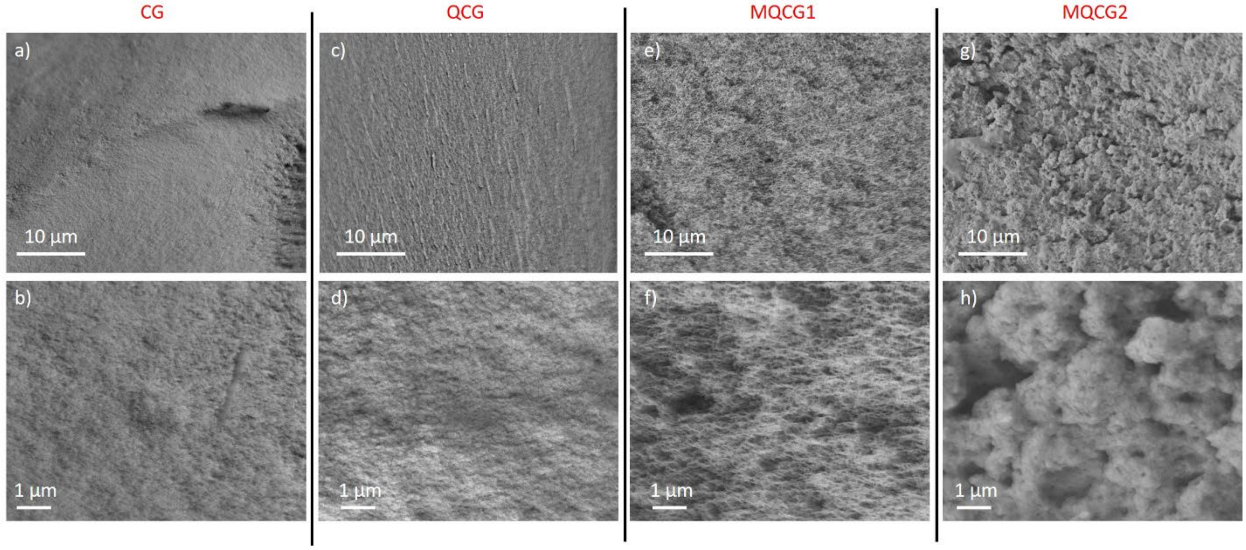

Figure S3: Cross-sectional SEM images of the hydrogels at two magnifications: (a, b) CG at ×2000 and ×10000, respectively; (c, d) QCG at ×2000 and ×10000; (e, f) MQCG1 at ×2000 and ×10000; (g, h) MQCG2 at ×2000 and ×10000.

Figure S3 illustrates the structure of the hydrogels' cross-sections at two different magnifications. The cross-sectional surface of the MQCG1 samples **(Fig. S3e–f**) demonstrates that sacrificial templating using 1 wt.% PEG6000 blended with chitosan resulted in the formation of internal pores, in contrast to the CG (**Fig. S3a–b**) and QCG (**Fig. S3c–d**) samples. The CG and QCG samples exhibit smoother internal surfaces with little or no pores, confirming the lower BET surface area results reported in the manuscript (Section 2.1.5). MQCG2 (**Fig. S3g–h**) displays a highly porous internal structure, corresponding to a significantly higher surface area compared to

MQCG1. The lower porosity in MQCG1 compared to MQCG2 is attributable to the lower content of PEG6000 removed from the hydrogel structure during the sacrificial templating procedure.[4]

4. **Isotherm and Kinetics of Adsorption Studies**

To evaluate the adsorption behavior of CGs, QCGs, and MQCGs, equilibrium isotherm studies were performed by exposing a fixed amount of each adsorbent to aqueous solutions of the target adsorbate at varying initial concentrations (1, 10, 50, 250, 500, 1000, and 5000 μg/L) under constant temperature. The equilibrium data were analyzed using the linear forms of the Langmuir and Freundlich isotherm models. The Langmuir isotherm, which assumes monolayer adsorption onto a homogeneous surface, was expressed as:

$$\frac{C_e}{q_e} = \frac{1}{K_L q_{max}} + \frac{C_e}{q_{max}} \tag{3}$$

Where C_e $({}^{mg}/_{L})$ is the equilibrium concentration of the adsorbate, q_e $({}^{mg}/_{g})$ is the amount adsorbed at equilibrium, q_{max} $({}^{mg}/_{g})$ is the maximum monolayer adsorption capacity, and K_L $({}^{L}/_{mg})$ is the Langmuir constant.[5]

The Freundlich isotherm, suitable for describing adsorption on heterogeneous surfaces, was applied in its linear form:

$$log q_e = \log K_F + \frac{1}{n} log C_e \tag{4}$$

Where K_F $({}^{mg}/_{g}\,({}^{L}/_{mg})^{\frac{1}{n}})$ and n are Freundlich constants related to adsorption capacity and intensity, respectively. The model parameters were determined by plotting the respective linear equations and evaluating the regression coefficients (R^2) to assess the best fit for each adsorbent type.[6]

To investigate the adsorption kinetics of hydrogels, time-dependent adsorption experiments were conducted by contacting a fixed dose of adsorbent (10 mg) with a known concentration of the target adsorbate solution (40 mL of 500 μg/L PFOS) under controlled agitation (40 rpm) at room temperature. Samples were withdrawn at time intervals (1, 2, 5, 10, 20, 30, …,2880 minutes), and the residual adsorbate concentrations were analyzed to determine the amount adsorbed at time t, denoted as q_t (mg/g). The experimental kinetic data were evaluated

using the linear forms of the pseudo-first-order and pseudo-second-order models. The pseudo-first-order model, which assumes the rate of occupation of adsorption sites is proportional to the number of unoccupied sites, was applied using the equation:

$$\text{Log}(q_e - q_t) = \log q_e - \frac{K_1}{2.303} t \quad (5)$$

Where q_e and q_t ($^{mg}/_{g}$) are the adsorption capacity at equilibrium and at time t (min), respectively, and k_1 ($^{1}/_{min}$) is the pseudo-first-order rate constant.

The pseudo-second-order model, based on the assumption that chemisorption is the rate-limiting step, was expressed as:

$$\frac{t}{q_t} = \frac{1}{K_2 q_e^2} + \frac{t}{q_e} \quad (6)$$

where K_2 ($\frac{g}{mg}.min$) is the pseudo-second-order rate constant. The kinetic parameters were determined from the slope and intercept of the respective linear plots, and the correlation coefficient (R^2) was used to assess the applicability of each model in describing the adsorption process for the different hydrogels.[7]

5. Effect of Quaternization and Surface Modification on Zeta-Potential

The resulting graphs from zeta-potential analysis through DLS is presented in **Figure S4**.

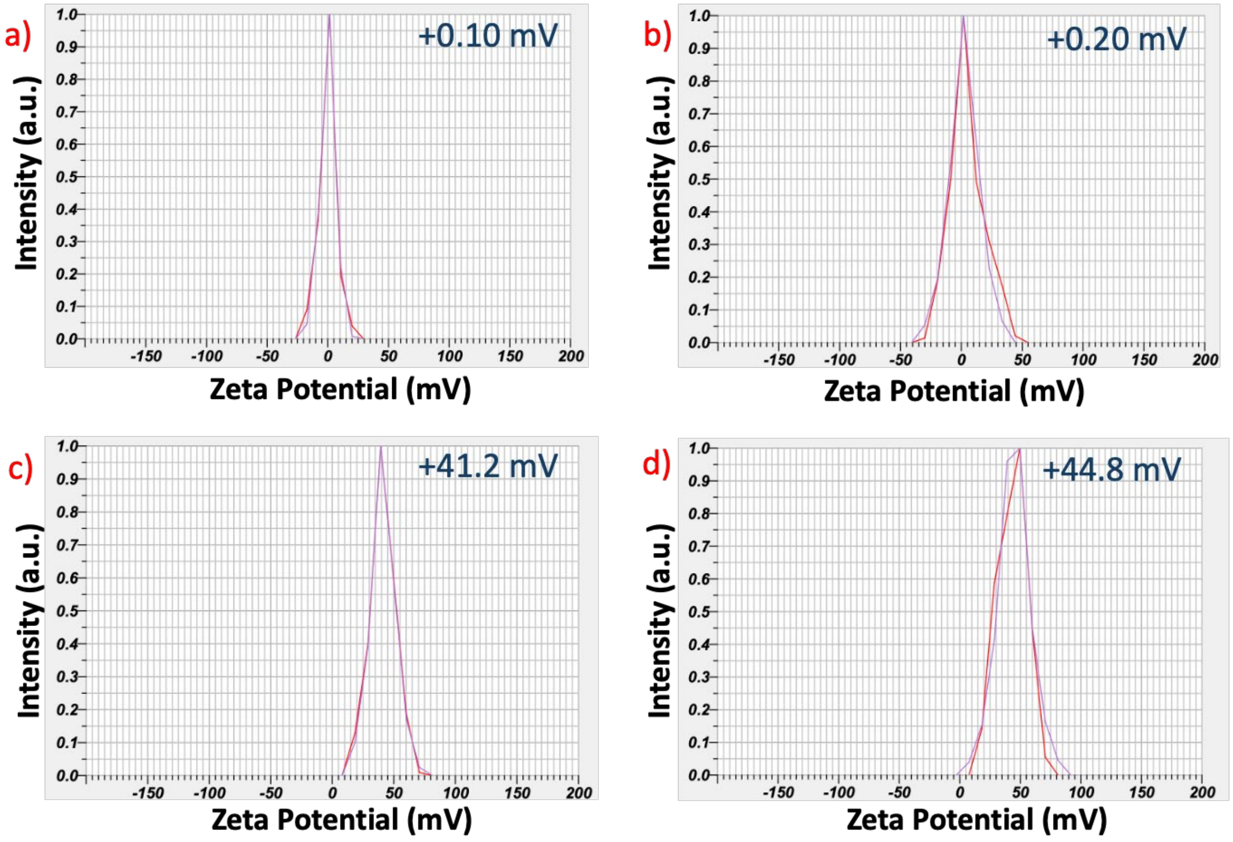

Figure S4: Zeta-Potential graphs of a) Chitosan powder, b) Chitosan hydrogel (CG) c) Quaternized Chitosan hydrogel, d) Surface-modified quaternized Chitosan hydrogel.

References:

1. Wu, Z.; Zhang, P.; Zhang, H.; Li, X.; He, Y.; Qin, P.; Yang, C. Tough Porous Nanocomposite Hydrogel for Water Treatment. *J. Hazard. Mater.* **2022**, *421*, 126754, doi:10.1016/j.jhazmat.2021.126754.
2. Wang, C.; Zhou, Y. Sacrificial Biomaterials in 3D Fabrication of Scaffolds for Tissue Engineering Applications. *J. Biomed. Mater. Res. B Appl. Biomater.* **2024**, *112*, e35312, doi:10.1002/jbm.b.35312.
3. Aigoin, J.; Payré, B.; Minvielle Moncla, J.; Escudero, M.; Goudouneche, D.; Ferri-Angulo, D.; Calmon, P.-F.; Vaysse, L.; Kemoun, P.; Malaquin, L.; et al. Comparative Analysis of Electron Microscopy Techniques for Hydrogel Microarchitecture Characterization: SEM, Cryo-SEM, ESEM, and TEM. *ACS Omega* **2025**, *10*, 14687–14698, doi:10.1021/acsomega.4c08096.
4. Heimbuck, A.M.; Priddy-Arrington, T.R.; Sawyer, B.J.; Caldorera-Moore, M.E. Effects of Post-Processing Methods on Chitosan-Genipin Hydrogel Properties. *Mater. Sci. Eng. C* **2019**, *98*, 612–618, doi:10.1016/j.msec.2018.12.119.
5. Zhou, D.; Brusseau, M.L.; Zhang, Y.; Li, S.; Wei, W.; Sun, H.; Zheng, C. Simulating PFAS Adsorption Kinetics, Adsorption Isotherms, and Nonideal Transport in Saturated Soil with Tempered One-Sided Stable Density (TOSD) Based Models. *J. Hazard. Mater.* **2021**, *411*, 125169, doi:10.1016/j.jhazmat.2021.125169.
6. Shahrokhinia, A.; Tafazoli, S.; Rijal, S.; Shuster, D.B.; Scanga, R.A.; Morefield, D.J.; Garay, J.; Rocheleau, R.A.; Bagheri Kashani, M.; Nagarajan, R.; et al. Dynamic Worm-Gel Materials as Tunable, Regenerable Adsorbents for Water Treatment. *Macromolecules* **2024**, *57*, 628–639, doi:10.1021/acs.macromol.3c02090.
7. Pranić, M.; Carlucci, L.; Van Der Wal, A.; Dykstra, J.E. Kinetic and Isotherm Study for the Adsorption of Per- and Polyfluoroalkyl Substances (PFAS) on Activated Carbon in the Low Ng/L Range. *Chemosphere* **2025**, *370*, 143889, doi:10.1016/j.chemosphere.2024.143889.
